# Antidiabetic agent use and clinical outcomes in patients with diabetes hospitalized for COVID-19: a systematic review and meta-analysis

**DOI:** 10.3389/fendo.2024.1482853

**Published:** 2025-01-06

**Authors:** Jordan N. Keels, Isabella R. McDonald, Christopher S. Lee, Andrew A. Dwyer

**Affiliations:** ^1^ Boston College, William F. Connell School of Nursing, Boston, MA, United States; ^2^ P50 Massachusetts General Hospital, Harvard Center for Reproductive Medicine, Boston, MA, United States

**Keywords:** diabetes, COVID-19, antidiabetic agents, inpatient care, diabetes management, patient outcomes

## Abstract

**Background:**

The effect of antidiabetic agents on mortality outcomes is unclear for individuals with diabetes mellitus (DM) who are hospitalized for COVID-19.

**Purpose:**

To examine the relationship between antidiabetic agent use and clinical outcomes in individuals with DM hospitalized for COVID-19.

**Methods:**

A systematic review of the literature (2020-2024) was performed across five databases. Included articles reported primary research (English) reporting clinical outcomes of adult patients (≥18 yrs.) with DM receiving antidiabetic agents who were hospitalized for COVID-19. Following PRISMA guidelines articles underwent independent dual review. Quality appraisal was completed for included studies. Independent reviewers used a structured data extraction form to retrieve relevant data. Aggregated data were synthesized by treatment regimen and reported descriptively. Random effects meta-analyses were performed to assess relative risk and prevalence of mortality.

**Results:**

After removing duplicates, title and abstract screening of 4,898 articles identified 118 articles for full-text review and 35 articles were retained for analysis. Included articles were primarily from China (15/35, 43%) and retrospective in nature (31/35, 89%). Fourteen studies (40%) assessed multiple antidiabetic agents, fifteen studies (42%) focused on metformin, three studies (9%) assessed the use of DPP-4 inhibitors, and three single studies (9%) investigated the use of insulin, TZD, and SGLT2 inhibitors. Despite differences among studies, the overall relative risk of mortality among metformin and DPP-4 inhibitor users was 0.432 (95% CI = 0.268-0.695, z = 3.45, p < 0.001) and the overall prevalence of mortality among all antidiabetic users was 16% (95% CI = 13%–19%, z = 10.70, p < 0.001).

**Conclusions and implications:**

Synthesis of findings suggest that patients who remained on oral agents (with/without supplemental insulin therapy) exhibited decreased mortality and lower inflammatory markers. Results indicate that individuals with DM should continue oral antidiabetic agents with additional basal insulin as needed to improve glycemic control and reduce mortality. Further work is needed to uncover mechanism(s) and clarify medical management approaches.

## Background

Severe acute respiratory syndrome coronavirus 2 (SARS-CoV-2) is a non-segmented, enveloped, positive-strand RNA virus causing a global pandemic of COVID-19 (1). COVID-19 is known to cause wide-ranging clinical complications, most notably respiratory failure. A number of studies have reported a close interaction between COVID-19 and metabolic disruption including diabetes mellitus (DM) (1). Type 1 diabetes mellitus (T1DM) is considered to be autoimmune in etiology and is characterized by insulin deficiency resulting from destruction of pancreatic ß-cells (2). Type 2 diabetes mellitus (T2DM) is characterized by persistent elevated blood glucose concentrations (hyperglycemia) in the setting of insulin resistance and hyperinsulinemia and accounts for nearly 90% of DM cases worldwide (3). Recent literature demonstrates a bidirectional relationship between diabetes and COVID-19 (4). Notably, a recent systematic review and meta-analysis the prevalence of DM increases with COVID-19 severity, and diabetes accounts for 9.5% of severe COVID-19 cases and 16.8% of COVID-19 related deaths (5). Importantly, a 2022 systematic review and meta-analysis of 10 articles spanning 11 patient cohorts (47.1 million total individuals) analyzed the relative risk of incident DM in patients with COVID-19. Investigators found a 64% greater risk of incident DM in patients with COVID-19 compared to non-COVID-19 infected controls (6).

A 2021 study showed patients with DM who tested positive for COVID-19 have an increased risk for intensive care unit (ICU) admission (17.6% vs. 7.8%) and mortality (20.3% vs 10.5%) compared to patients without DM who tested positive for COVID-19 (7). The degree of hyperglycemia appears to modulate COVID-19 severity, and a higher hemoglobin A1c (HbA1c) is associated with increased mortality (8). Despite an overall decline in mortality rates among individuals with COVID-19, poor outcomes persist among individuals with comorbid chronic disease (9). A retrospective study of 453 hospitalized patients that evaluated the association between the degree of hyperglycemia and the risk of all-cause mortality among hospitalized patients with COVID-19 found that patients with pre-existing DM or new-onset DM were more likely to experience complications, including acute kidney injury, compared to COVID-19 patients with normoglycemia (15.3-17.0% vs 1.5-3.1%), hypoalbuminemia (36.7% - 39.4% vs 10.8-19.4%), acute respiratory distress syndrome (ARDS) (3.1%-10.5% vs 0.8-3.1%) and severe COVID-19 complications (i.e., kidney disease, ischemic heart disease) (82.7-89.4% vs 61.4-72.15) (10).

Antidiabetic agents have both anti-inflammatory and immunomodulatory effects (11). Numerous studies, including systematic reviews and meta-analyses, have evaluated the effect of antidiabetic agents and mortality rates among individuals with DM (9). To date, the effect of antidiabetic agents on patient outcomes in hospitalized individuals with a dual diagnosis of DM and COVID-19 remains unclear. This study aimed to explore the relationship between different antidiabetic drug classes and patient outcomes among patients with DM who are hospitalized for COVID-19.

## Methods

We conducted a systematic review of existing literature on outcomes for patients who were hospitalized for COVID-19. The review was registered in Prospero (CRD42023476297). Findings are reported according to the Preferred Reporting Items for Systematic Reviews and Meta-Analyses (PRISMA) guidelines (12).

### Literature search

We conducted a comprehensive, systematic literature search of five databases (PubMed, Embase, CINAHL, Web of Science, and Cochrane Library) on February 12, 2023 that was conducted again on September 27, 2024 to capture articles published since the initial literature search. A Boolean search was performed using the following key words/MeSH terms: “COVID-19” or “COVID” or “SARS-CoV-2 or “coronavirus” or “2019 novel coronavirus disease” AND “diabetes” or “diabetes type 1” or “diabetes type 2” or “new-onset diabetes” or “diabetes mellitus” or “newly diagnosed diabetes” AND “antidiabetic agents” or “metformin” or “insulin” or “ sodium-glucose cotransporter 2 inhibitors (SGLT2 inhibitors)” or “dipeptidyl peptidase IV inhibitors (DPP-4 inhibitors)” or “ thiazolidinediones (TZD)” or “sulfonylurea compounds” or “glucagon-like peptide 1 agonists (GLP-1 agonists)”. No restrictions were applied to the search. The retrieved articles were imported into EndNote20 (13). Additional full-text articles were identified by hand searching the reference lists of relevant articles (i.e., snowball approach).

### Study selection

**Figure 1:**
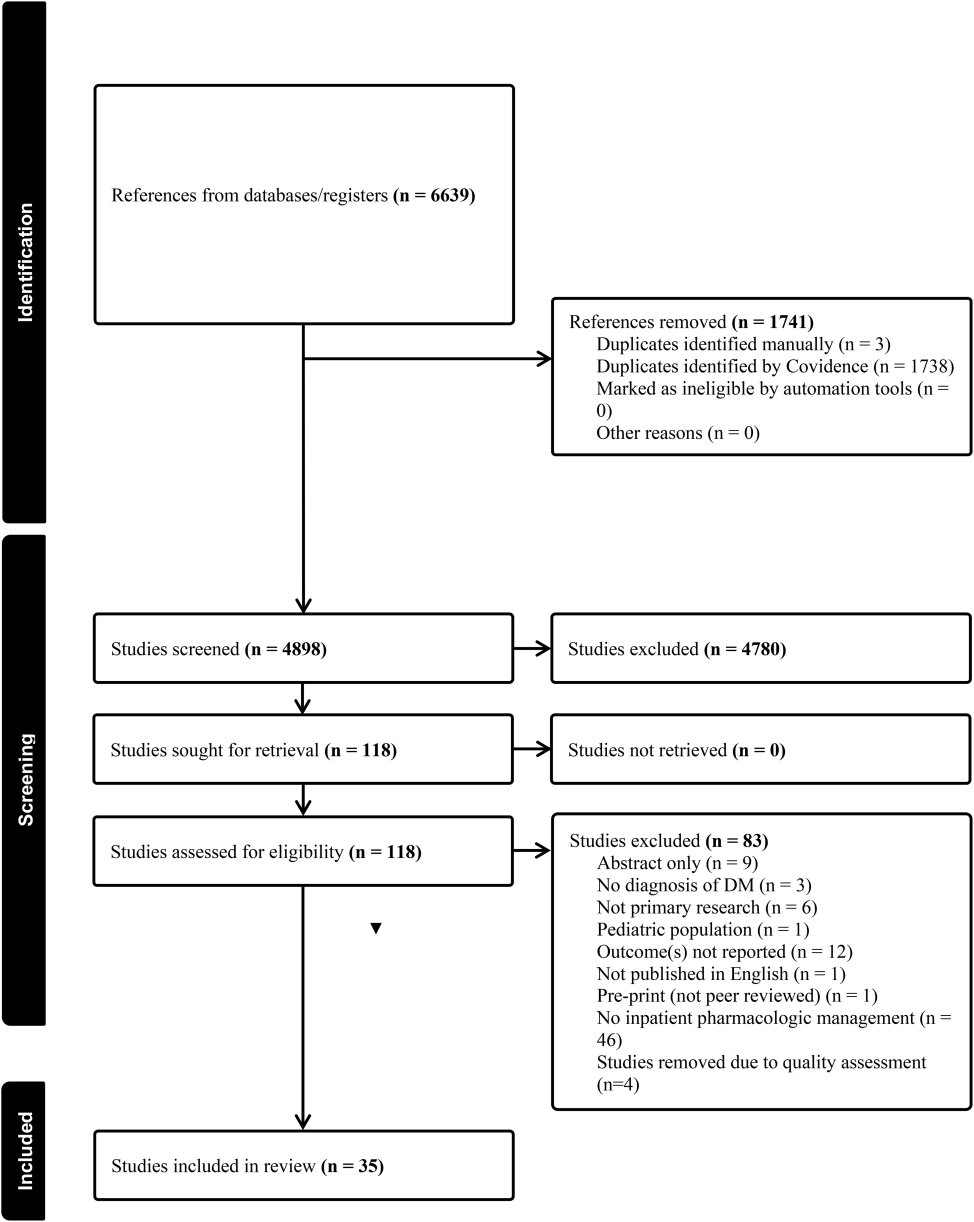
PRISMA diagram.

We employed the PRISMA guidelines (12) to inform article selection. Articles meeting the following inclusion criteria were included for analysis: (1) primary research articles; (2) written in English; (3) published in a peer-reviewed journal; (4) evaluating clinical outcomes of adult patients (18+ yrs.) with DM who were hospitalized for COVID-19; (5) receiving antidiabetic agents or drug classes (i.e., metformin, insulin, SGLT2 inhibitors, DPP-4 inhibitors, GLP-1 agonists, a TZD, or sulfonylurea). Articles published before 2020 as well as letters to the editor, reviews, commentaries, medical hypotheses, abstracts, editorials, theses, and dissertations were excluded. Similarly, studies not reporting participant diagnosis and/or outcomes or studies reporting animal studies or *in vitro* models were not included. Two independent investigators (JNK, IRM) conducted title and abstract screening followed by the full-text screening. Disagreements were decided by discussion with the third investigator (AAD). We used Covidence systematic review management software (14) throughout the review process. **Figure 1** depicts the PRISMA diagram detailing the study selection process.

### Data extraction

Two independent reviewers (JNK, IRM) used predetermined categories to extract relevant data including title, first author surname, publication year, country and setting, study design, study aim, sample size, participant demographics (age, gender, nationality), reported outcomes, and limitations. Extracted data were collated in an evidence table (**Supplementary Materials**). The Joanna Briggs Institute Critical Appraisal tool (https://jbi.global/critical-appraisal-tools) was used to assess the methodological quality of included studies. After dual quality appraisal raw data and sample sizes were extracted from relevant studies reporting on mortality. To ensure a comprehensive analysis missing data was directly solicited from corresponding authors.

### Analysis

The primary outcome of the analysis was mortality. Secondary outcomes included incidence of acidosis, relevant inflammatory biomarkers (lactate dehydrogenase [LDH], creatine kinase-MB [CK-MB], D-dimer), incidence of acute respiratory distress syndrome (ARDS), time to clinical improvement, length of stay, and intensive care admission rates.

To quantitatively examine overall relative risk and prevalence of mortality, random-effects meta-analyses were performed (15). Prevalence is based on a binomial distribution, representing the ratio of events to non-events within the sample or between exposure and control groups. The overall relative risk and prevalence of mortality was quantified by the incidence reported in the studies. Accuracy of aggregate estimates is represented by 95% confidence intervals (CI), Z-scores and p-values as precision metrics signifying against the null hypothesis that there is no effect or no difference on mortality. Heterogeneity was quantified in this meta-analysis for the overall estimate. Total dispersion in effect sizes across studies (Q) and the associated p-value were calculated. I² was calculated to describe a fraction of the variance due to heterogeneity.

## Results

The search strategy yielded a total of 6,639 articles for review. Removing duplicates left 4,898 articles for title and abstract screening (**Figure 1**). The interrater reliability between the first and second coder, represented by the Cohens k coefficient, was 0.68 indicating substantial agreement (16). One hundred and eighteen articles were included for full-text review, Cohens k coefficient was 0.94, indicating substantial agreement (16). Of the articles retrieved for full-text review, 83 articles were excluded for not meeting study inclusion criteria (i.e. no inpatient pharmacologic management (n=46), outcomes not reported (n=12), abstract only (n=9), not primary research (n=6), poor quality (n=4), no diagnosis of DM (n=3), pediatric population (n=1), not published in English (n=1) and pre-print (not peer-reviewed n=1). A total of 35 articles were retained for analysis. The included studies reported research from 14 different countries. Notably, many of the studies originated from China (15/35, 42.8%) (11, 17–30). The remaining 20 studies were from the U.S.: n=3 (31–33), Iraq: n=3 (34–36), Israel n=2 (37, 38), Italy: n=2 (39, 40), Philippines: n=1 (41), Turkey: n=2 (42, 43), Brazil: n=1 (44), Indonesia: n=1 (45), Iran: n=1 (46), Denmark: n=1 (47), Poland: n=1 (48), Qatar and Kuwait: n=1 (49) and multinational=1 (50).

The majority of studies (31/35, 89%) used a retrospective design (11, 17–33, 35, 36, 38–45, 47, 48, 50). Three studies reported findings from a randomized controlled trial (RCT) (31, 37, 49) and one prospective cohort study (34). Cumulatively, included studies reported on 31,766 participants. Only one study from the U.S. included data on participant self-reported racial and ethnic identity (32). Diagnosis of COVID-19 was confirmed by polymerase chain reaction (PCR) testing or chest CT. Most studies (30/35, 85.7%) included individuals with T2DM (11, 17–28, 31, 32, 34–38, 40–43, 45–50). Two studies (2/35, 5.7%%) included both T1DM and T2DM (33, 44), and three studies (3/35, 8.6%%) did not specify diabetes type (29, 30, 39).

### Evaluation of multiple drug classes and patient outcomes

Fourteen retrospective studies (China (n=7), USA (n=1) Israel (n=1), Iraq (n=1), Turkey (n=1) Denmark (n=1), Poland (n=1), multi-national (n=1), assessed the relationship between multiple antidiabetic agents on patient outcomes (**Table 1**) (18, 25–31, 36, 38, 42, 47, 48, 50). Twelve (86%) studies evaluated individuals with T2DM (18, 25–28, 31, 36, 38, 42, 47, 48, 50), while two studies did not specify diabetes type (29, 30). Twelve (86%) studies specifically addressed mortality (18, 25, 27–30, 38, 42, 47, 48, 50). Among the studies reporting mortality data, four identified an association between insulin use and increased mortality (18, 25, 30, 38). In contrast, three studies found no significant association between insulin use and mortality (31, 47, 48). However, the risk of death was affected when other factors (i.e., age and C-reactive protein) were considered (48). Decreased mortality was linked to the use of metformin, alpha-glucosidase inhibitors, meglitinides, TZDs, SGLT2 inhibitors, and DPP-4 inhibitors (27–29). Notably, one study highlighted reduced mortality with GLP-1 agonists and the combined use of GLP-1 agonists, pioglitazone, or DPP-4 inhibitors, while an increased mortality risk was associated with the use of DPP-4 inhibitors alone (50). Two articles reported decreased mortality with oral agents alone and/or in combination with insulin (18, 28). One study reported on the use of metformin noting a lower risk of death and reduced incidence of ARDS (18). In addition, two articles found no significant association between oral antidiabetic agents and mortality (31, 42). One study reported the need for mechanical ventilation was higher among those receiving insulin (18). Articles investigating alpha-glucosidase inhibitors observed a decrease in ICU admissions (26, 29) and a reduced need for mechanical ventilation (26). One study reported higher incidence of ICU admission in patients treated with insulin compared to other antidiabetic agents (metformin, alpha-glucosidase inhibitors, sulfonylureas, and DPP-4 inhibitors) (25). Two studies examining numerous agents metformin, DPP-4 inhibitors (31), GLP-1 agonists, insulin, SGLT2 inhibitors and sulfonylureas noted no specific association between glucose lowering agents and ICU admission (47). Metformin use was associated with reduced disease severity, improved oxygenation, and enhanced glycemic control (36). One study found no significant association between metformin use and disease severity (29). Moreover, the use of metformin was linked to a decrease in the inflammatory biomarker D-dimer (36), while other studies reported increased neutrophil count and decreased lymphocytes with the use of metformin (30).

**Table 1:** All antidiabetic treatments.

Ref #	Diabetes type	Study Sample	Country	Treatment Modality
(18)	T2DM	n=53,030	China	Insulin, metformin, alpha-glycosidase inhibitors, sulfonylureas, glinides, and DPP-4 inhibitors
(25)	T2DM	n=689	China	Metformin, Alpha-glucosidase inhibitors, sulfonylureas, DPP-4 inhibitors, insulin-sensitizing agents
(26)	T2DM	n=52	China	Insulin, Alpha-glucosidase inhibitors, Metformin, DPP-4 inhibitors, TZD, Sulfonylurea secretagogue, non-sulfonylurea secretagogue
(27)	T2DM	n=131	China	Insulin, metformin, sulfonylureas, Alpha-glucosidase inhibitor (acarbose)
(28)	T2DM	n=108	China	Insulin, metformin, Alpha-glucosidase inhibitors, meglitinides, TZD, SGLT2 inhibitors, DPP-4 inhibitors
(29)	Unknown	n=64	China	Metformin, insulin, Alpha-glucosidase inhibitor (acarbose), TZD, sulfonylureas
(30)	Unknown	n=168	China	Insulin, oral antihyperglycemics (not specified)
(31)	T2DM	n=529	USA	Insulin, metformin, DPP-4 inhibitors
(36)	T2DM	n=112	Iraq	Metformin, sitagliptin
(38)	T2DM	n=359	Israel	Insulin, metformin, sulfonylureas, GLP-1, DPP-4 inhibitors
(42)	T2DM	n=432	Turkey	DPP-4 inhibitors, sulfonylureas, biguanides, SGLT2 inhibitors, TZD
(47)	T2DM	n=4,430	Denmark	DPP-4 inhibitors, GLP-1 agonists, insulin, metformin, SLGT-2 inhibitors, sulfonylureas
(48)	T2DM	n=430	Poland	Metformin, insulin
(50)	T2DM	n=29,516	Multinational*	DPP-4 inhibitors, GLP-1 agonists, TZD

DPP-4 inhibitors, Dipeptidyl peptidase 4 inhibitors; SGLT2 inhibitors, sodium-glucose cotransporter-2; TZD, Thiazolidinedione; GLP-1 agonists, Glucagon-like peptide 1 agonists; *TriNetX COVID-19 Research Network.

### Metformin and patient outcomes

Thirteen retrospective studies (11, 17, 20–24, 32, 35, 39, 41, 44, 45), one prospective cohort study (34), and one RCT (46) from eight countries (China (n=7), Iraq (n=2), Philippines (n=1), USA (n=1), Indonesia (n=1), Iran (n=1), Italy (n=1) and Brazil (n=1)) evaluated the use of metformin on patient outcomes. Majority of the studies assessed individuals with T2DM (11, 17, 20–24, 32, 34, 35, 41, 45, 46), one study included individuals with T1DM and T2DM (24) and one study did not specify (39). Eleven (73%) studies reported on the association between in-hospital metformin use and mortality. Eight studies found decreased mortality rates in individuals who continued metformin inpatient, compared to counterparts not on metformin groups (11, 17, 21, 23, 24, 27, 39, 41, 45). Two studies found no statistical difference between metformin and non-metformin users (44, 46). Furthermore, patients who were on metformin prior to hospitalization and discontinued metformin on admission had higher mortality rates compared to those who continued metformin therapy inpatient (44). One study reporting on ICU admissions showed an increased rate of ICU admissions with metformin use (20).

Overall, three studies reported biomarkers in patients with in-hospital metformin use noting decreased inflammatory and oxidative stress biomarkers (34) [D-dimer & LDH (22, 35), CRP (35) and CKMB (22)]. Furthermore, one study also noted decreased neutrophil counts and increased lymphocytes (21). In terms of metformin use and renal function, one study noted an increase in blood urea nitrogen (BUN) (22) and two studies reported an increased incidence of acidosis with metformin (21, 24). However, when adjusting for glomerular filtration rate (i.e., GFR>60) no difference was noted between metformin and non-metformin groups (21). Three studies examined metformin use and the development of ARDS, two identified decreased incidence of ARDS among metformin users (20, 21) and one reported increased incidence of ARDS among metformin users (11). Two studies reported reduced risk of need for mechanical ventilation among metformin users (17, 46). In addition, one study reported a decreased risk of respiratory failure and cardiac events with the use of metformin (17). Of the seven studies reporting on length of stay (17, 21–24, 32, 46), two reported a statistically significant association with shorter length of stay (17, 24).

### DPP-4 inhibitors and patient outcomes

Three studies from three different countries (Israel, Italy, China) evaluated the use of DPP-4 inhibitors on patient outcomes (19, 37, 40). Two were retrospective in nature (19, 40) and one was an RCT (37). All three studies included individuals with T2DM (19, 37, 40). Three (100%) studies reported on the association between in hospital DPP-4 inhibitor use and mortality. Two studies (67%) found no significant association between mortality and DPP-4 inhibitor use (19, 37) while one study (33%) found a decrease in mortality (40). Two studies (67%) reported on ICU admission; one (33%) noting a reduced risk for the need of the ICU (40), in contrast to the other noting no significant difference (37). One study (33%) reported a decline in inflammatory biomarkers (PCT and CRP) and an increase in lymphocytes (40). One study (33%) reported on the incidence of ARDS and acidosis noting no significant difference when compared to non-DPP-4 inhibitor groups (19).

### Insulin, TZD, SGLT2 inhibitors and patient outcomes

One retrospective, multicenter, observational study investigated the relationship between insulin use and patient outcomes among patients with both T1DM and T2DM (33). Study findings indicated that higher insulin doses were associated with greater mortality. A RCT conducted in Qatar and Kuwait investigated the association between pioglitazone and on a number of outcomes in patients with T2DM including need for mechanical ventilation, myocardial damage, mortality, inflammatory response, length of stay and development of acute coronary syndrome (49). Findings showed no significant clinical benefit of pioglitazone in patients with diabetes hospitalized for COVID-19 (49). One retrospective study examined the effect of dapagliflozin use on cardiovascular outcomes in patients with T2DM (43). The study reported lower rates of ICU admission and mortality among dapagliflozin users (43).

### Quantitative synthesis of mortality across all drug classes

**Figure 2:**
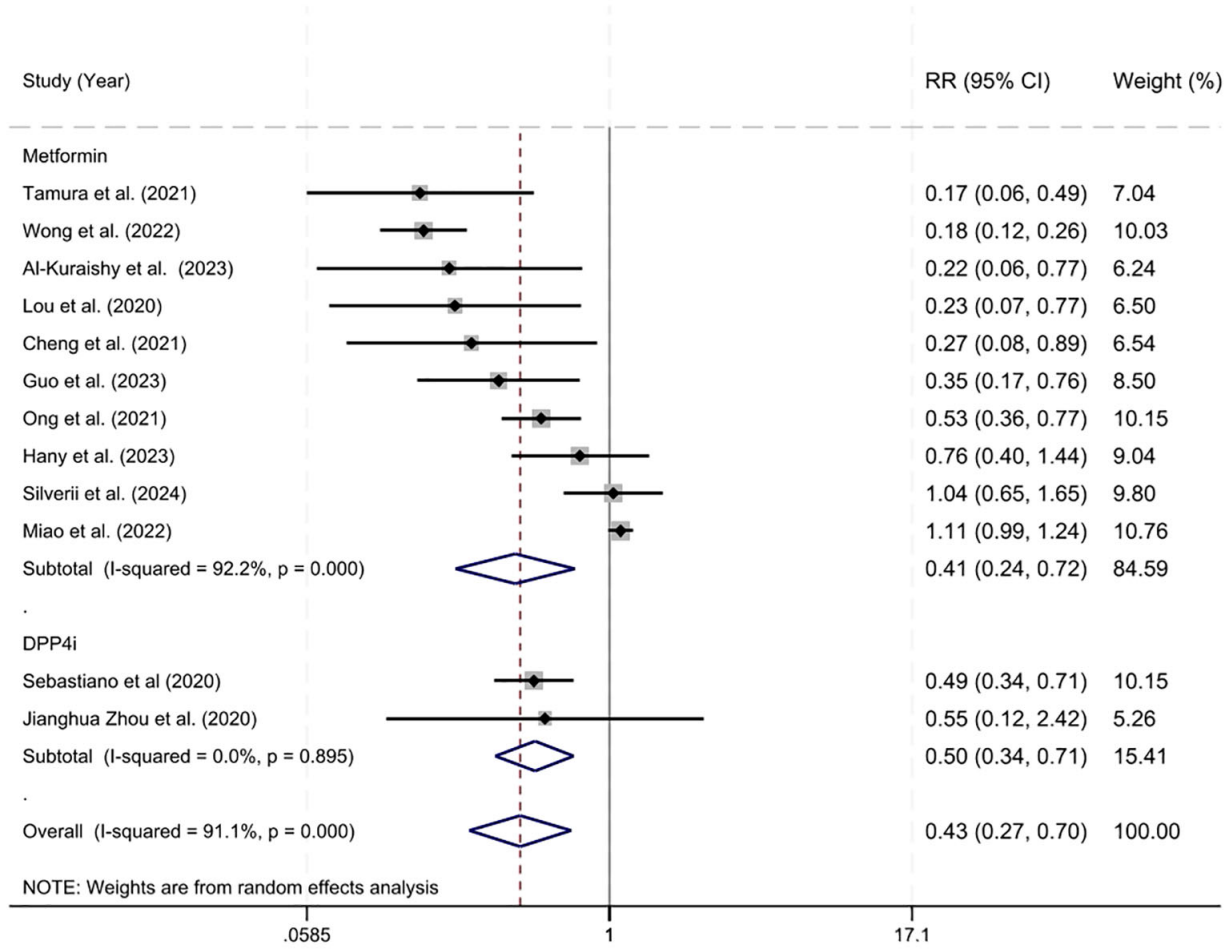
Estimated relative risk of mortality in metformin and DPP-4 inhibitor users.

Studies reporting crude data on mortality rates were included in an overall analysis (**Figure 2**). Across studies examining metformin and DPP-4 inhibitors the overall risk ratio of 0.432 (95% CI = 0.268-0.695, z = 3.45, p < 0.001) indicating a 57% risk reduction among individuals using either metformin or DPP-4 inhibitors compared with control treatment. There was considerable variability in effects across studies (Q = 123.83, *df* = 11, p < 0.001, I^2^ = 91.1%) meaning mortality outcomes varied significantly among studies. The predictive interval ranged from 0.27 to 0.70. Effects sizes also varied between metformin and DPP-4 inhibitors (Q= 8.80, *df* = 1, p=0.003) indicating that drug choice is not equally effective in preventing mortality. Insulin was not included due to potential risk of bias related to advanced diabetes and the associated confounding by indication.

**Figure 3:**
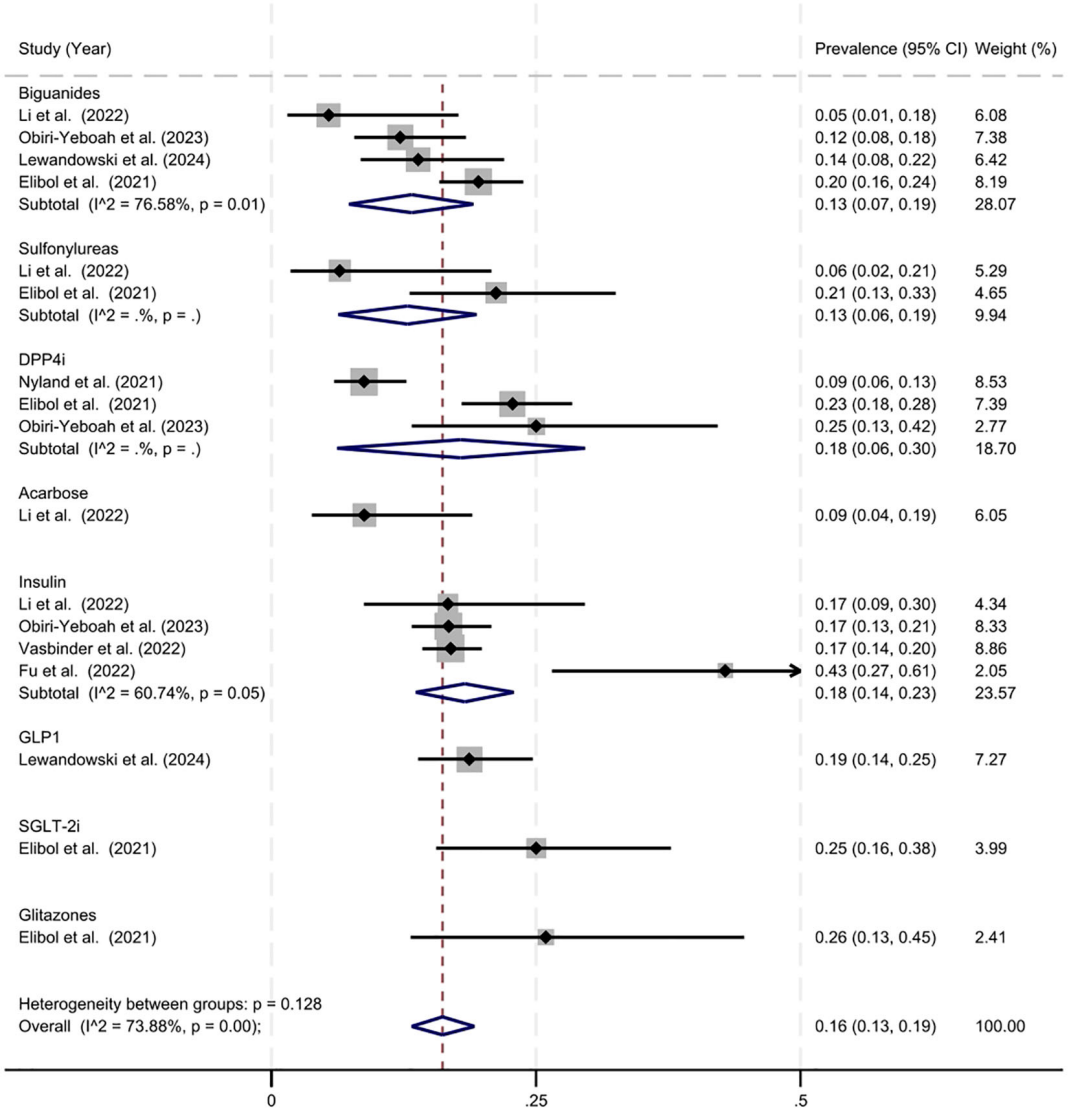
Estimated prevalence of mortality in antidiabetic agent users.

The overall estimated prevalence of mortality in individuals using antidiabetic agents was 16% (95% CI = 13%–19%, z = 10.70, p < 0.001) (**Figure 3**). Heterogeneity statistics (Q = 61.24, *df* = 16, p = < 0.001, I^2^ = 73.88%) indicating significant and substantial variance in the prevenance of mortality across studies. The estimated prevalence of mortality with biguanide use was 13% (95% CI = 7%–19%, z = 4.44, p < 0.001). The estimated prevalence of mortality with sulfonylureas was similar at 13% (95% CI = 6%–19%, z = 3.88, p < 0.001). The estimated prevalence of mortality with DPP-4 inhibitor use was slightly higher at 18% (95% CI = 6%– 30%, z = 3.00, p < 0.001). The estimated prevalence with insulin was similar at 18% (95% CI = 14%–23%, z = 7.72, p < 0.001). The remaining drug classes (acarbose, GLP-1 inhibitors, SGLT-2 inhibitors, and glitazones included only one study, thus they should be interpreted cautiously.

Three studies (18, 21, 47) reported Cox proportional hazard ratios of mortality across drug classes (insulin, sulfonylureas, DPP4-inhibitors, metformin, GLP-1 inhibitors, SGLT-2 inhibitors, alpha-glucosidase inhibitors and glinides) was 0.948 (95% CI = 0.695–1.200, Q = 53.15, *df* = 12, p = < 0.001, I^2^ = 77.4%). The estimated hazard ratio was highest with insulin use 1.535 (95% CI = 0.507–2.563) and lowest with alpha-glucosidase inhibitors 0.530 (95% CI = 0.325–0.735).

## Discussion

We report findings from a systematic review of the literature on antidiabetic agents and outcomes among patients with DM hospitalized for COVID-19. We synthesized findings from 35 included articles reporting on different antidiabetic agents: metformin, alpha-glucosidase inhibitors, meglitinides, TZD, SGLT2 inhibitors, DPP-4 inhibitors, and insulin. A summary of the two predominant outcomes (i.e. mortality and ICU admission) can be found in **Table 2**.

**Table 2:** Summary of findings on antidiabetic medication and mortality and ICU admission.

Agent	Mortality	ICU
Metformin	n=12	n=1
	Decreased mortality (11, 17, 18, 21, 23, 24, 27, 39, 41, 45) No association (44, 46)	Increased ICU admissions (20)
Alpha-glucosidase inhibitor	n=4	n=2
	Decreased mortality (26–29)	Decreased ICU admission (26, 29)
GLP-1 agonist	n=1	
	Decreased mortality (50)	
DPP-4 inhibitors	n=3	n=2
	Decreased mortality (37, 40, 50)	Decreased ICU admissions (40) No association (37)
SGLT2 inhibitors	n=1	n=1
	Decreased mortality (43)	Decreased ICU admission (43)
TZD	n=1	n=1
	No association (49)	No association (49)
Insulin	n=5	n=2
	Increased mortality (18, 25, 28, 30, 33)	Increased ICU admission (25, 31)
Oral agents + Insulin	n=1	
	Decreased mortality (42)	
Oral agents*	n=2	n=1
	No association (42, 47)	No association (47)

*DPP-4 inhibitors, sulfonylureas, biguanides, SGLT2 inhibitors, thiazolidinediones, GLP-1 agonists.

A preliminary review of studies using the JBI tool identified high quality studies suitable for a meta-analysis on the risk and prevalence of mortality among antidiabetic agent users. Overall, our findings suggest a reduced relative risk of mortality among metformin and DPP-4 inhibitor users (RR = 0.432 (95% CI = 0.268-0.695, z = 3.45, p < 0.001)). This is consistent with findings in previous meta-analyses examining mortality risk with metformin use among antidiabetic users (51–58) and DPP-4 inhibitors (52, 59, 60). The overall prevalence of mortality was 16% (95% CI = 13%–19%, z = 10.70, p < 0.001).

Fourteen studies reported on the relationship between different antidiabetic agents and patient outcomes (18, 25–31, 36, 38, 42, 47, 48, 50). Studies were primarily retrospective in nature (n=14), conducted in China (n=7) and assessed individuals with T2DM (n=12). Twelve of the studies assessed antidiabetic use and mortality noting a decrease in mortality with the use of oral antidiabetic agents (metformin, acarbose, a-glucosidase inhibitors, meglitinides, TZD, SGLT2 inhibitors and DPP-4 inhibitors) (27–29). Conversely, an increase in mortality was associated with insulin use (18, 25, 30, 38). However, when insulin was combined with oral agents a decrease in mortality was reported (28). An increase in mortality with insulin use is consistent with previous studies (52). The underlying mechanism behind this phenomenon is unclear (52). However, from a clinical perspective, insulin is often given during late-stage DM suggesting other comorbidities may be contributing to increased mortality. This is further supported by increased rates of ICU admission with insulin use (25) in contrast to a decrease in ICU admission with alpha-glucosidase inhibitors (26, 29) and other antidiabetic agents (metformin, alpha-glucosidase inhibitors, sulfonylureas, and DPP-4 inhibitors) (25).

Metformin was associated with reduced disease severity, improved oxygenation, better glycemic control and decreased D-dimer (36). Previous studies have reported on the anti-inflammatory and antiviral effects of metformin, suggesting they may have protective mechanisms in individuals with COVID-19 (61). Similar to studies reporting on all diabetic agents, studies reporting on in-patient metformin use were primarily retrospective in nature and conducted in China. In parallel to previous findings, studies evaluating metformin use reported decreased mortality (11, 17, 20–24, 39, 45). Recent data suggests that there is an increase in oxidative stress in individuals with T2DM and COVID-19 increasing the risk of inflammatory and coagulation disorders (62). However, when metformin was used a decrease in inflammatory biomarkers (D-dimer, ferritin, LDH, and CRP) was reported (22, 34, 35).

Metformin has demonstrated the ability to impede the attachment of SARS-CoV-2 to ACE2 receptors by stimulating the phosphorylation of ACE2 (63). Additionally, it has been shown to modulate the immune response by fostering the generation of anti-inflammatory regulatory T cells and macrophages (63). Moreover, metformin suppresses the activation of inflammatory signaling pathways implicated in COVID-19 (64). These findings suggest that metformin could possess anti-inflammatory and antioxidative properties, potentially mitigating the robust inflammatory response and oxidative stress associated with SARS-CoV-2 infection (65). As a result, these effects may elucidate the observed reduction in inflammatory biomarkers. Furthermore, recent literature has reported that metformin may be beneficial in reducing long-COVID (66). Our study also noted a decreased incidence of ARDS (20, 21) and reduced risk for cardiac and respiratory events (17). However, one study noted an increase in incidence of ARDS (11) suggesting more research needs to be done to gain a better understanding of the role of metformin and incidence of ARDS. Three studies reported a significant relationship between metformin and length of stay - noting a reduced duration of hospitalization (17, 24, 46). Suggesting that patients who remained on metformin may have better outcomes when compared to non-metformin users.

Three studies evaluated individuals with T2DM and reported on the use of DPP-4 inhibitors and patient outcomes. Our analysis demonstrated a reduced risk of mortality with DPP-4 inhibitor use. However, there were only a few studies assessing this relationship, thus further research needs to be conducted to fully understand the association between the two. Previous studies have suggested that DPP-4 is a ubiquitous glycoprotein that could act as both a cell membrane protein and soluble enzymatic protein after cleavage and release. Recent data suggests that DPP-4 inhibitors could alter the outcomes in individuals positive for COVID-19 infection and alter oxygen requirements through their effects on the cardiovascular system (67). However, our findings suggest a varied results regarding DPP-4 inhibitors with one study reporting a decline in ICU admission (40), in contrast to one reporting no significant difference (37). Only one study reported on inflammatory biomarkers noting a reduction in PCT and CRP (40). Furthermore, no significant difference was found when evaluating ARDS or acidosis. Our findings on DPP-4 inhibitors are limited to only a few studies, future research needs to be done to determine underlying mechanisms contributing to patient outcomes in individuals diagnosed with DM and COVID-19.

One study focused solely on the use of insulin and patient outcomes, as previously noted increased mortality was associated with insulin use. While insulin is the recommended treatment for individuals hospitalized with DM (68), our findings suggest combining insulin with an oral antidiabetic agent may have positive effects on patient outcomes in patients with DM hospitalized for COVID-19. One study examined the effect of pioglitazone on T2DM hospitalized for COVID-19, however no significant findings were identified (49). Such findings are similar to previous literature examining drug efficacy on mortality (69). Last, one study examined dapagliflozin use in individuals with DM hospitalized for COVID-19 and their study reported lower ICU rates and reduced mortality among users (43). Tisch et al., conducted a systematic review examining the potential use of SGLT2 inhibitors in patients with acute illness (70). Findings are similar noting a reduced risk for mortality, however in individuals with prior use of SGLT2 inhibitors there was no association with ICU admission (70). We recognize that viewing individual medications is one dimensional and these outcomes are likely related to many other factors, such as disease severity, diabetes duration, age, comorbidities, medications and SARS-CoV-2 variant. Therefore it is important that further studies investigate the interactions between antidiabetic medications and other potential confounding factors.

A relative strength of this study is the comprehensive and systematic review and meta-analyses of the literature and synthesis of current evidence providing insight into clinical outcomes in patients with DM hospitalized for COVID-19. It merits noting that this work has several limitations. First, most (n=31/35, 88.6%) included articles reported on retrospective studies. In addition, most studies lacked detailed data on patient demographics, and some did not specify the type of diabetes. Furthermore, the meta-analyses were limited by data reported in studies and information solicited from corresponding authors. It is also important to note that the studies did not provide detailed information on the variant underlying COVID-19. This data may also be impacted by the strain of COVID-19, recent literature reports a decline in the number of individuals with DM with evolving variants (71). Additionally, patient outcomes are also contingent upon the care and inpatient management. The findings on insulin and patient outcomes should be interpreted with caution. For example, many patients are often taken off their outpatient medications and placed on insulin during hospitalization in line with current American Diabetes Association (ADA) recommendations (72). The ADA guidelines generally recommend insulin as the preferred treatment during inpatient hospital admission, with resumption of outpatient medications following discharge (72). Notably, patients on insulin may have more severe DM or require tighter glycemic control during acute illness (e.g., COVID-19). As the pandemic progressed, understanding of the sequelae grew and clinicians gained valuable experience providing improved management. Thus, as clinical practice advanced, outcomes were likely affected. So, comparing outcome data from early days of the pandemic (i.e., Wuhan strain) with data collected on hospitalizations secondary to subsequent variants (i.e., Delta, Omicron variants) is challenging. Moreover, most studies were from China, so further research is warranted to assess patient outcomes across varied health systems as diabetes management may vary across countries and health finance structures.

## Conclusions

Synthesizing the findings from the systematic review and meta-analyses reveals that metformin use and DPP-4 inhibitor use are associated with decreased mortality in individuals with DM who were hospitalized for COVID-19. Data also suggest that insulin treatment is associated with increased mortality rate. However, our findings may relate to other patient-level factors including age, comorbidities, SARS-CoV-2 variant, severity of COVID-19, and duration of diabetes. COVID-19 induces a potent inflammatory response. It is plausible that the anti-inflammatory properties of metformin may confer a protective role for patients with DM who are hospitalized for COVID-19. The beneficial effects of metformin and DPP-4 inhibitors suggest that patients who are established on these antidiabetic agents should remain on their current treatment (i.e., not changed to insulin) if hospitalized for COVID-19. Given the changing landscape of COVID-19 and the emergence of new variants, further studies and randomized control trials are needed to confirm and validate these findings.
